# Identification of compounds from natural Peruvian sources as potential inhibitors of SARS-CoV-2 Mpro mutations by virtual screening and computational simulations

**DOI:** 10.12688/f1000research.143633.1

**Published:** 2024-04-04

**Authors:** Haruna Luz Barazorda-Ccahuana, Eymi Gladys Cárcamo Rodriguez, Angela Emperatriz Centeno-Lopez, Margot Paco-Chipana, Luis Daniel Goyzueta-Mamani, Miguel Angel Chavez-Fumagalli

**Affiliations:** 1 Computational Biology and Chemistry Research Group, Vicerrectorado de Investigación, Universidad Catolica de Santa Maria de Arequipa, Pedro Vilcapaza, Arequipa, 04000, Peru; 2 Facultad de Ciencias Farmaceuticas, Bioquímicas y Biotecnológicas, Universidad Catolica de Santa Maria de Arequipa, Pedro Vilcapaza, Arequipa, 04000, Peru; 3 Sustainable Innovative Biomaterials, Le Qara Research Center, Arequipa, Peru

**Keywords:** Main protease, mutations, SARS-CoV-2, Peruvian sources, rutin

## Abstract

**Background:**

The coronavirus disease (COVID-19) pandemic continues to be a public health problem worldwide. Several therapeutic targets of the severe acute respiratory syndrome coronavirus 2 (SARS-CoV-2) have been identified, whereas the main protease (Mpro) is necessary for virus replication. Since SARS- CoV-2 Mpro mutation rates are inherently high, searching for new inhibitors remains challenging. Herein, this work aimed to evaluate 84 natural compounds from Peruvian sources against different mutations on the Mpro target.

**Methods:**

We applied virtual screening, all-atom molecular dynamics simulations, and binding free energy estimation by Molecular Mechanics/Generalized Born Surface Area (MM/GBSA).

**Results:**

The virtual screening results helped us identify rutin as the top compound against different Mpro mutations. Likewise, the computational simulations demonstrated the high structural stability of the Mpro-rutin system.

**Conclusions:**

his research evaluated the antiviral capacity of Peruvian sources against SARS-CoV-2 Mpro and its mutations, which could be important in preventing and treating SARS-CoV-2 infection.

## Introduction

At the end of 2019, the world observed the outbreak of the coronavirus disease (COVID-19) pandemic, which began to spread within communities and hospitals, causing numerous persons to become infected. ^1^ Severe acute respiratory syndrome coronavirus 2 (SARS-CoV-2) is responsible for triggering the pandemic known as COVID-19 ^2^^,^^3^ and until today, the virus transmission is wreaking on public health ^4^ and the world economy. ^5^ Several countries had reported variants of SARS-CoV-2, and by the end of the second half of 2020, they had rapidly spread. ^6^^,^^7^

The SARS-CoV-2 main protease (Mpro) is a key enzyme that plays a fundamental role in mediating viral replication and transcription. ^8^ Mpro processes the polyprotein 1ab at multiple cleavage sites and hydrolyses the Gln-Ser peptide bond in the Leu-Gln-Ser-Ala-Gly recognition sequence. This cleavage site in the substrate is distinct from the peptide sequence recognized by other human cysteine proteases known to date. ^9^ It is also considered a potential therapeutic target since its inhibition could help block the virus’s translation and replication. ^10^ Mpro comprises three domains: domains I, II, and III, composed of 8-101, 102-184, and 201-306 amino acid residues, respectively. ^8^

Likewise, several researchers had highlighted the importance of studying the stability of Mpro structure taking to account the mutations, since it would be challenging to identify specific inhibitors. ^11^ These variants are characterized by changes in the amino acid sequence of the virus when compared with the first sequenced strain Wuhan-Hu-1 (Gen- Bank accession: NC_ 045512.2); the variants may have one or more mutations that differentiate them from the wild type. ^12^ The SARS-CoV-2 genetic variations are crucial to track and evaluate their spread in countries. It must be considered that the registered mutations can change the binding mechanisms of possible inhibitors developing a potential resistance. ^13^ Therefore, it is crucial to anticipate the effect and identify new inhibitors that counteract these effects. In the absence of a specific drug and the appearance of new mutations, different studies evaluate the potency of many phytochemicals to restrict the multiplication of SARS-CoV-2 and other viral infections. ^14^ Phytocompounds might be the most promising drug candidate in the current need since they have high bioavailability and low toxicity. ^15^ Likewise, *in silico* studies have shown the potent inhibitory action against SAR-CoV-2 Mpro of taraxerol from *Clerodendrum* spp. used in traditional medicine in Asia tropical regions ^16^ and the *β*-amyrin and stigmasta-5,22-dien-3-ol present in *Cyperus rotundus L.* used in traditional medicine in India. ^17^

Peru is one of the 12 nations with the largest percentage of biodiversity, and because of this, a living legacy of traditional medicine has been able to grow and endure over time. ^18^ The Vavilov Institute views this region as a global hub for floral biodiversity. ^19^ The 20,000–30,000 plant species found in the various locations make up roughly 10% of all the plants used as medicine worldwide. ^20^

This study used the Peruvian Natural Products Database (PeruNPDB) dataset for docking-based virtual screening. Subsequently, the best compound was analyzed by molecular dynamics simulations and binding free energy estimation from eight SARS-CoV-2 Mpro mutated (Y54C, ^21^ N142S, ^21^ T190I, ^21^ A191V, ^21^ S139A, ^22^ R298A, ^22^ R60C, ^13^ and G11A ^23^).

## Methods

### Proteins preparation

The SARS-CoV-2 Mpro crystal structure was selected. To sample the mutations, they were obtained of the native sequence only for the Mpro reported in the Protein Data Bank by the access code PDB ID: 5RE4, which each amino acid was replaced for eight mutations in search of new challenges (R298A, N142S, A191V, R60C, G11A, Y54C, T190I, and S139A). The preparation of mutated systems was done by homology modeling in the SWISS-MODEL server using the crystal structure of SARS-CoV-2 Mpro (PDB ID: 5RE4) as a template.

### Preparation of the virtual database and screening

The search for natural products was performed at the Peruvian Natural Products Database (PeruNPDB) online web server (first version) (accessed on 23 January 2022), ^24^ whereas the simplified molecular-input line-entry system (SMILE) of each compound of was the upload into OpenBabel within the Python Prescription Virtual Screening Tool (PyRx) ^25^; and the subjection to energy minimization; whereas PyRx performs structure-based virtual screening by applying docking simulations using the AutoDock Vina tool. ^26^ Likewise, the FASTA sequence of the Crystal Structure of SARS-CoV-2 main protease (Mpro) (PDB: 5RE4) was subjected to a BLAST search (accessed on 16 April 2022), ^27^ whereas all the mutants were selected and subjected to automated modeling in SWISS-MODEL server (accessed on 17 April 2022). ^28^ For the analysis, the search space encompassed the whole of the modeled 3D models; and the docking simulation was then run at exhaustiveness of eight and set to only output the lowest energy pose. Multiple sequence alignments of the Mpro and mutant sequences were visualized using the msa package (version 1.22.0) ^29^ in the R programming environment (version 4.0.3). The heatmap plot was generated using GraphPad Prism version 9.4.0 (673) for Windows, GraphPad Software, San Diego, California USA.

### Molecular dynamics simulation and Molecular Mechanics/Generalized Born Surface Area (MM/GBSA) calculation

The simulation of the motion is realized by the numerical solution of the classical Newtonian dynamic equations. ^30^ We used Gromacs v. 2020 to calculate the molecular dynamics (MD) simulation and the AMBER-99SB-ILDN force field. The topologies for the Amber force field were determined on the ACPYPE server for the best metabolite against mutates Mpro. Each system was included in the center of a cube box of 10 on each side. Likewise, water molecules were added (water model TIP4P). The energy minimization was carried out with the steep-descendent integrator with 200,000 calculation steps. Herein, the MD simulation in the canonical ensemble NVT was done for a time of 1 ns. Finally, the production of MD continued 100 ns in the isobaric-isothermal ensemble considering the Parrinello-Rahman barostat (1 bar) and V-rescale thermostat (309.65 K). The binding free energy estimation by MM/GBSA (Molecular Mechanics/Generalized Born Surface Area) was calculated with the suit mmpbsa.py ^31^ from AmberTools20 ^32^ and gmx MMPBSA v1.4.1. ^33^ The equations related to calculations of binding free energies are the following:

$${∆G}_{\text{bind}}=G_{\text{complex}}-(G_{\text{protein}}+G_{\mathrm{lig}})$$
^(1)^

$$={\text{∆E}}_{M\;M}+{\text{∆G}}_{\mathrm{GB}}+{\text{∆G}}_{\mathrm{SA}}-T∆S$$ (2)

$$={\text{∆E}}_{\mathrm{vdw}}+{\text{∆E}}_{\mathrm{ele}}+{\text{∆G}}_{\mathrm{GB}}+{\text{∆G}}_{\mathrm{SA}}-T∆S$$ (3)

The equation that determines the electrostatic solvation energy ( *∆G _GB_*) considers ( *∆E _M M_*) which is the variation between the minimized energy of the protein-ligand complexes of the study which includes the van der Waals ( *∆E _vdw_*) and electrostatic ( *∆E _ele_*) contributions, while ( *∆G _SA_*) is the difference in surface area energies for protein and ligand and ( *T∆S*) refers to the contribution of entropy at temperature *T*.

Finally, the graphical visualizations were made with Visual Molecular Dynamics (VMD), ^34^ allowing interactive visualization with an easy-to-use interface. The interpretation of the molecular interactions was recreated with Maestro (Schrodinger) 2D interactions diagram. Likewise, the Molecular dynamics simulation results were performed by the Gromacs tools, and the values were processed by Gnuplot 5.2 command-driven interactive function plotting program.

## Results

### Mutant SARS-CoV-2 Mpro description

SARS-CoV-2 Mpro is a cysteine protease of 67.6 kDa, and its structure possesses a catalytic dyad (Cys145 and His41) with a substrate-binding pocket located in a cleft between domains I and II. The secondary structure of Mpro has 10 alpha helixes, 13 beta sheets, and eight beta protrusions, seven beta hairpins, 22 beta turns, five gamma turns, and nine helix-helix interactions. In this work we used the access code PDB ID: 5RE4, which was downloaded from the Protein Data Bank. This crystal structure was determined by the X-ray diffraction method with a resolution of 1.88 Å.

**Figure 1.**
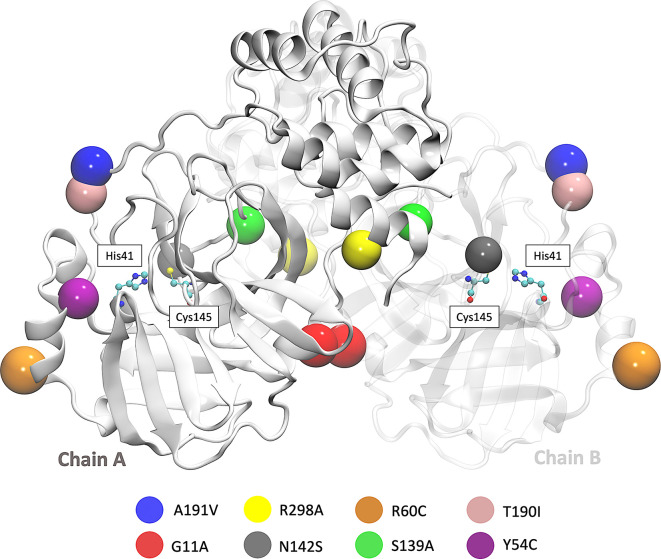
3D representation of SARS-CoV-2 Mpro in which the eight mutations are located. The beads represent the location of each mutation on both Mpro chains. SARS-CoV-2, severe acute respiratory syndrome coronavirus 2; Mpro, main protease.

We also focused on the analysis of eight mutations registered in different parts of the world. First is the Y54C mutation reported in Malaysia, and the N142S mutation was reported 17 times in five different countries. T190I is a mutation identified in 15 countries, such as South Africa and the USA. The mutation A191V is characterized by having an occurrence rate of 0.30% and is present in more than 34 countries. Besides, in the S139A, G11A, and R298A, ^35^ mutations result in the complete loss of dimerization. ^36^ In Brazil and Vietnam, the R60C mutation was reported, affecting the protein dynamics and the inhibitor’s binding within its active site. ^13^ The R298A leads to the interruption of the dimeric conformation and irreversible inhibition of the enzyme’s catalytic activity, ^36^ and the G11A mutation avoids the insertion of the N finger region (residues 1-9) and therefore wholly declines its activity. ^37^ The location of the eight mutations is shown in Figure 1. ^45^

**Figure 2.**
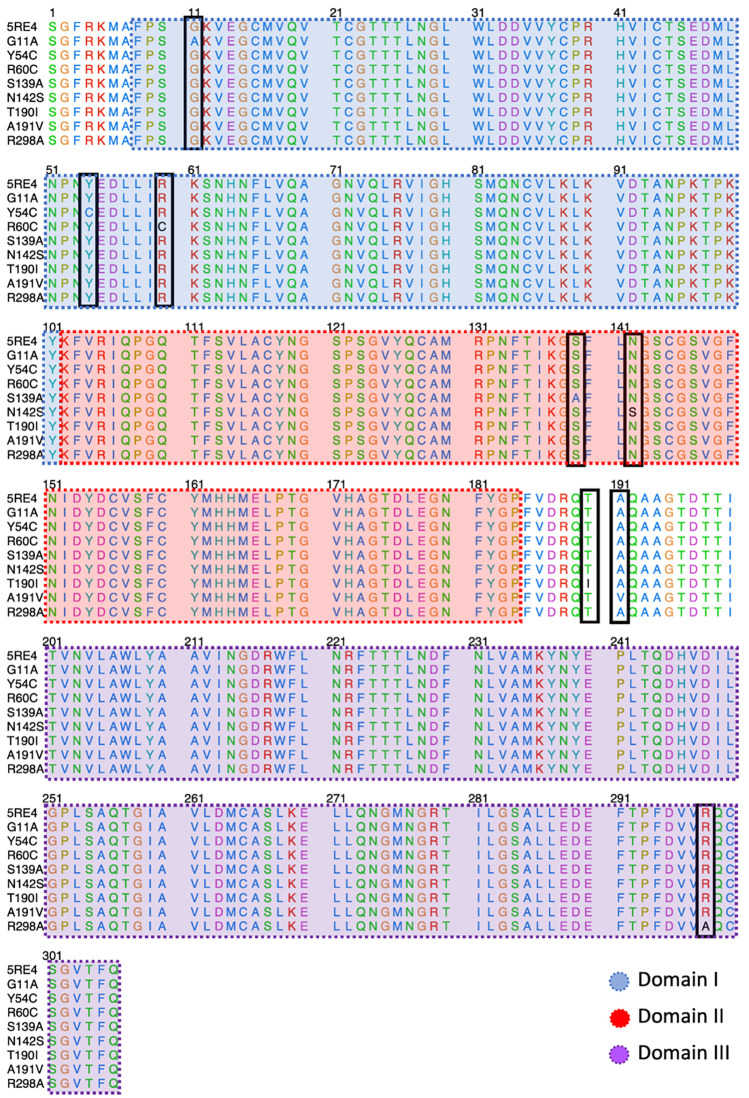
Sequence alignment of SARS-CoV-2 Mpro with the different proteins mutated. The blue, red, and purple boxes represent Domain I, Domain II, and Domain III, respectively. SARS-CoV-2, severe acute respiratory syndrome coronavirus 2; Mpro, main protease.

Figure 2 shows the sequence alignment of Mpro mutations. The black square selects the variation of residues by mutant Mpro. The G11A, Y54C, and R60C mutations are located close to the His41 residue and in Domain I from Mpro. Two mutations (S139A and N142S) are present in the Domain II and close to the Cys145, and it is expected that these protein structures could show different behavior than Mpro without mutations. On the other hand, it was also observed that mutations in T190I and A191V are in the connection of Domain II and Domain III. For the case of R298A mutation, it can be observed near Domain III.

### Virtual screening analysis

The virtual screening technique, widely used for drug discovery, seeks to identify potential compounds for a particular therapeutic target. This approach allowed us to find new possible candidates within the PeruNPDB dataset against one of the therapeutic targets from mutant Mpro of SARS-CoV-2.

**Figure 3.**
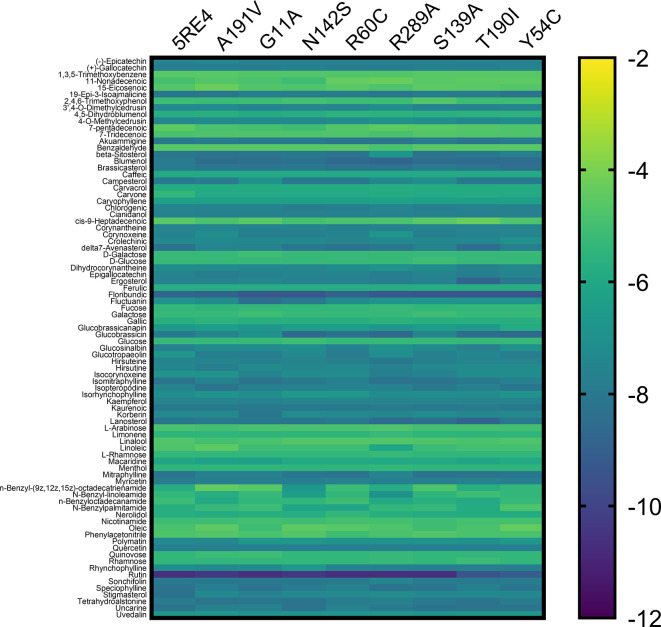
Heat map analysis of binding constants of metabolites from Peruvian native plants screened against mutated Mpro of SARS-CoV-2. SARS-CoV-2, severe acute respiratory syndrome coronavirus 2; Mpro, main protease.

The 84 substances included in the study are taken from the original PeruNPDB collection; the most recent dataset consists of 280 substances. Figure 3 shows the gradient palette, the violet color indicated strong binding ( *∆*G<-12 kcal/mol), while the yellow color indicated weak binding (∆G>-2 kcal/mol). In this heat map, rutin is shown the best compounds. However, for the T190I and Y54C mutations, the color intensity is lower compared to the other mutations.

In Table S1 of the underlying data, ^45^ the values of coupling energies are reported. The values for Mpro wild, A191V, G11A, N142S, R60C, R289C, S139A, T190I, and Y54C were -10.7 kcal/mol, -10.4 kcal/mol, -10.7 kcal/mol, -10.4 kcal/mol, - 10.7 kcal/mol, -10.7 kcal/mol, -10.7 kcal/mol, -9.4 kcal/mol, and -9.1 kcal/mol, respectively.

### Molecular dynamics simulations and estimation of binding free energy

**Figure 4.**
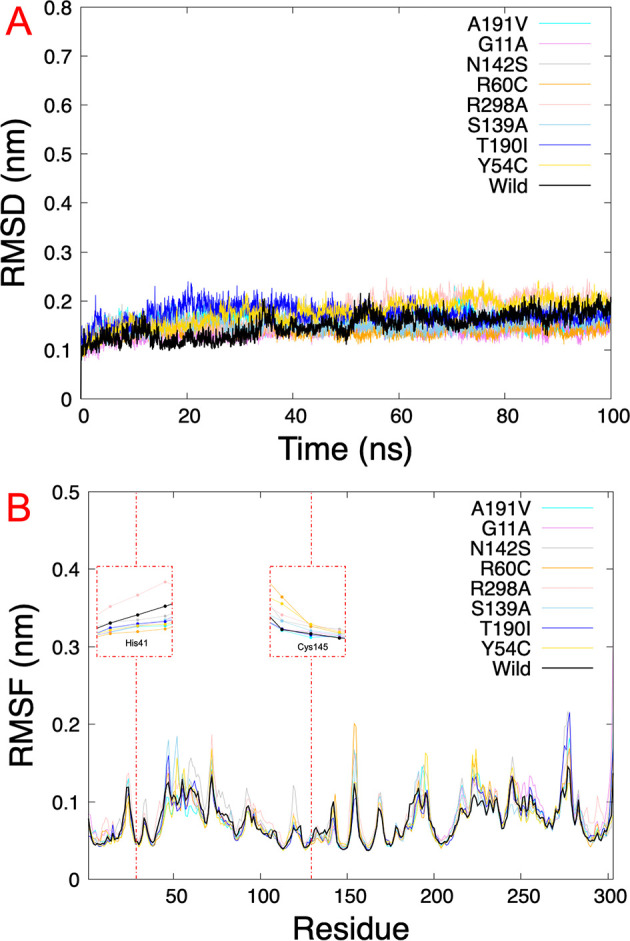
RMSD and RMSF plots. A.) RMSD of eight SARS-CoV-2 Mpro with rutin. B.) RMSF of the last 5 ns *per residue* of each SARS-CoV-2 Mpro mutated, highlighting the principal residues of the catalytic dyad (His41 and Cys145). RMSD, Root-mean square deviation; RMSF, Root-mean square fluctuation; SARS-CoV-2, severe acute respiratory syndrome coronavirus 2; Mpro, main protease.

The results obtained from virtual screening helped us to consider rutin as a ligand against the different Mpro mutations. Molecular dynamics simulations allow us to understand the behavior of different mutated Mpro at an atomistic level. After analyzing 100 ns of production dynamics, the convergence of each protein is observed by Root-mean squared deviation (RMSD) analysis (see Figure 2A). This result shows us that the different types of mutations achieved equilibrium; likewise, an average RMSD between 0.1 and 0.2 nm is appreciated, an acceptable value in this structural model. The Root-mean squared fluctuation (RMSF) calculates the flexibility of individual residues that make up the Mpro protein during a simulation trajectory. The RMSF *per residue* diagram structurally indicates which amino acids in a protein contribute the most to a molecular motion. Figure 4B highlights the area of His41 and Cys145 amino acids where the most significant fluctuation in the His41 area occurs with the R298A mutation, while the most significant fluctuation in the Cys145 area occurred in the R60C, Y54C, R298A, and N142S mutation.

On the other hand, Table 1 shows us the quantitative values of the RMSD, where the G11A and R60C mutations showed the lowest average RMSD value. By contrast, the average value for the Y54C mutation was higher than the others. Regarding the average RMSF values of the last 5 ns, for the 5RE4, A191V, G11A, N142S, R60C, S139A, and T190I systems, the average RMSF of the Mpro structures oscillated by 0.8 nm, while for R298A the RMSF average results in 0.09 nm and Y54C it was 0.07 nm.

**Table 1.** RMSD and RMSF average values of SARS-CoV-2 Mpro wild and mutated.

System	RMSD (nm)	RMSF (nm)
5RE4	0.15 *±*0.02	0.08 *±*0.03
A191V	0.16 *±*0.02	0.08 *±*0.03
G11A	0.14 *±*0.02	0.08 *±*0.04
N142S	0.16 *±*0.02	0.08 *±*0.03
R60C	0.14 *±*0.01	0.08 *±*0.03
R298A	0.17 *±*0.03	0.09 *±*0.03
S139A	0.15 *±*0.02	0.08 *±*0.03
T190I	0.17 *±*0.02	0.08 *±*0.03
Y54C	0.18 *±*0.03	0.07 *±*0.03

RMSD, Root-mean square deviation; RMSF, Root-mean square fluctuation; SARS-CoV-2, severe acute respiratory syndrome coronavirus 2; Mpro, main protease.

The results of the binding free energy estimation were performed by the MM/GBSA method with all frames of the MD. Table 2 indicates the average free energy values for each system. The values show a high coupling energy estimate, indicating that the interaction was carried out correctly.

**Table 2.** Calculated Molecular Mechanics/Generalized Born Surface Area (MM/GBSA) binding free energy of the systems.

System	∆ TOTAL	VDWAALS	EEL	EGB	∆G gas	∆G solv
5RE4	-33.49±7.32	-44.09±6.48	-33.36±16.91	50.02±9.38	-77.45±14.88	43.96±9.18
A191V	-40.71±6.07	-47.19±4.43	-43.39±10.75	55.74±7.21	-90.58±12.04	49.88±7.03
G11A	-41.17±4.48	-46.51±3.29	-42.57±10.84	53.40±7.96	-89.08±10.55	47.91±7.78
N142S	-36.65±3.82	-54.18±3.16	-26.84±6.44	51.02±5.15	-81.02±6.77	44.37±5.10
R60C	-45.09±7.29	-48.90±5.88	-49.45±12.23	59.24±7.93	-98.35±13.21	53.26±7.10
R298A	-24.11±8.41	-36.30±8.91	-21.09±12.85	37.81±12.91	-57.39±19.44	33.28±11.74
S139A	-25.84±2.95	-45.56±2.63	-18.41±7.38	43.84±5.14	-63.97±6.74	38.13±5.11
T190I	-35.87±5.80	-48.17±5.82	-30.99±8.97	49.32±5.99	-79.15±9.71	43.29±5.81
Y54C	-34.53±5.62	-28.04±5.52	-66.78±10.26	65.39±6.59	-94.82±10.55	60.29±6.36

VDWAALS = Van Der Waals energy; EEL = Electrostatic energy; EGB = Electrostatic contribution free energy calculated by Generalized Born; *∆*G gas = estimated binding free energy phase gas; *∆*G solv = estimates binding free energy solvent; *∆* TOTAL = Estimated binding free energy. Values of energy in kcal/mol.

The mutation R60C showed the best interaction energy (-45.09 kcal/mol) against the different systems studied. The energy values for G11A and A191V were -41.17 kcal/mol and -40.71 kcal/mol, respectively. While the systems that showed low binding energy were mutations R298A and S139A, with average values of -24.11 kcal/mol and -25.84 kcal/mol, respectively.

Likewise, the most significant energy contribution was given by the Van der Waals energies (VDWAALS) in the wild systems, A191V, G11A, N142S, R298A, S139A, and T190I. These types of energy are weak and are short-range interactions; in biological systems, they play a significant role in stabilizing protein-small molecules. On the other hand, in the R60C and Y54C systems, the energy contribution is given by electrostatic energies (EEL). Electrostatic energy takes into account the charges of each atom in the system, which depend on the medium in which they are found; these have greater scope, and the force of interaction it possesses is linked to the relative orientations it accepts.

**Figure 5.**
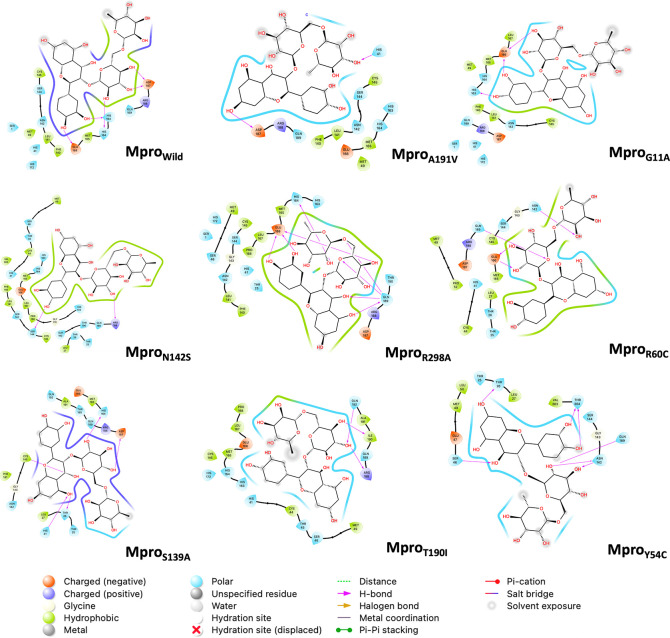
2D interaction diagram of rutin. The pink arrow lines represent the hydrogen bond. Mpro, main protease.

Figure 5 shows the last frame of each simulation of the Mpro-rutin complex. In general, it was observed that the interactions in the active site are due to the formation of hydrogen bonds. However, we observed some changes in the region around the active site for the mutations occurring in N142S and Y54C. The residues around N142S are mostly hydrophobic (green contour), hence N142S exhibits a greater energy contribution from hydrophobic interactions (VDWAALS = -54.18 kcal/mol, higher than the other mutations). While in Y54C, the residues around rutin are polar (sky blue contour), demonstrating its high energetic contribution by electrostatic interactions (EEL = -66.78 kcal/mol more elevated than the other mutations).

## Discussion

Until today, different research teams worldwide have collected information on SARS-CoV-2 strains since some show many mutations in the different structural proteins, like the main protease. Mutations affect the stability of the 306 residues of the main protease, giving them new characteristics. ^38^ Regarding other *in silico* studies carried out in sequences of SARS-CoV-2 Mpro mutated from India and Vietnam, they showed that this change in the genome affected the stability of the protein and impacted the catalytic zone. ^13^ For this reason, it is crucial to investigate how these mutations in SARS-CoV-2 Mpro can be inhibited and could prevent SAR-CoV-2 replication.

Different phytochemical molecules such as kaempferol, quercetin, luteolin-7-glucoside, demethoxycurcumin, naringenin, apigenin-7-glucoside, oleuropein, curcumin, catechin, and epicatechin gallate have been reported with promising antiviral drug against SARS-CoV-2. ^39^ Also, Parvez *et al.*, reported azobechalcone, rifampin, isolophirachalcone, tetrandrine, and fangchinoline as potential inhibitors of SARS-CoV-2 Mpro, ^40^ and Padhi *et al.*, who obtained that putaminoxin B, putaminoxin D, jasmonic acid, and jasmonic methyl ester with good pharmacokinetic properties against Mpro. ^41^ In 2020, more than a thousand FDA-approved drugs were virtually screened using molecular docking and binding free energy calculations, where nelfinavir was suggested as a potential inhibitor against SARS-CoV-2.

Goyzueta *et al.*, studied rutin compound as a promising inhibitor against the native SARS-CoV-2 Mpro by *in silico* techniques. ^42^ Likewise, reused drugs and phytochemical compounds showed a binding affinity against some Mpro mutants; for example, salvianolic acid A (extracted from *S. miltiorrhiza*) shows an inhibition effect against N142S and T190I. ^21^ The novelty of this work was to use a database of compounds designed with metabolites from Peruvian plants reported in the literature. Our results demonstrate that the rutin metabolite present in *S. sonchilofolius* (commonly known as yacón) and *L. meyenii* (commonly known as maca andina) had the best binding affinity with all proposed Mpro mutations. Rutin is known as rutoside and it is a natural phenolic compound with an essential role in the oxidant- antioxidant balance associated with some diseases. ^43^^,^^44^

Molecular dynamics simulation provides us with information on the structural stability of the protein at a given time by RMSD and RMSF analysis. Here the RMSD results were within an acceptable average in all systems, which indicates that the rutin molecule forms a highly stable complex. Additionally, the binding energy was also evaluated with the help of the MM/GBSA approach. In these, we have observed that the mutations located close to the active center are the ones that achieved the best energy contributions. In general, it was observed that all the Mpro-rutin systems have a high affinity.

## Conclusions

SARS-CoV-2 mutations have caused worry because some mutations can make the virus more aggressive and spread faster. Currently, several mutations have been reported in different therapeutic targets of SARS-CoV-2. The main protease (Mpro) is essential for SARS-CoV-2 replication and is a promising drug target. Here, we have focused on eight mutations of the SARS-CoV-2 Mpro (Y54C, N142S, T190I, A191V, S139A, R298A, R60C, and G11A) and analyzed several compounds from Peruvian natural sources by virtual screening methods, where rutin was the most suitable compound. Molecular dynamics simulations and binding free energy estimation by MM/GBSA showed high stability of the Mpro- rutin complex and excellent energetic affinity, respectively. These results demonstrated the database PeruNPDB’s utility in finding rutin as a promising inhibitor of different SARS-CoV-2 Mpro mutations.

## Ethics and consent

Ethical approval and consent were not required.

## Author contributions

Conceptualization: H.L.B.-C. and L.D.G.-M.; data curation: H.L.B.-C., L.D.G.-M., E.G.C.-R., A.E.C.-L., M.P.-C., and M.A.C.-F; formal analysis: H.L.B.-C. and M.A.C.-F.; funding acquisition: H.L.B.-C. and M.A.C.-F.; investigation: H.L.B.-C., M.P.-C., L.D.G.-M., E.G.C.-R., A.E.C.-L., and M.A.C.-F; methodology: H.L.B.-C. and M.A.C.-F.; writing—review and editing: H.L.B.-C., and M.A.C.-F. All authors have read and agreed to the published version of the manuscript.

## Data Availability

Figshare: Dataset of results from virtual screening and molecular dynamics simulations. https://doi.org/10.6084/m9.figshare.24271972. ^45^ This project contains the following underlying data:
1.
Table S1. (Virtual screening results of the compounds selected against SARS-COV2 Mprotein mutations). 2.
Table S2. (Molecular dynamics simulations results of the compounds selected against SARS-COV2 Mprotein mutations). 3.
Table S3. (Molecular dynamics simulations results of the compounds selected against SARS-COV2 Mprotein mutations). Data are available under the terms of the Creative Commons Attribution 4.0 International license (CC-BY 4.0).
