# Identification of compounds from natural Peruvian sources as potential inhibitors of SARS-CoV-2 Mpro mutations by virtual screening and computational simulations

**DOI:** 10.12688/f1000research.143633.3

**Published:** 2024-11-22

**Authors:** Haruna Luz Barazorda-Ccahuana, Eymi Gladys Cárcamo Rodriguez, Angela Emperatriz Centeno-Lopez, Margot Paco-Chipana, Luis Daniel Goyzueta-Mamani, Miguel Angel Chavez-Fumagalli

**Affiliations:** 1 Computational Biology and Chemistry Research Group, Vicerrectorado de Investigación, Universidad Catolica de Santa Maria de Arequipa, Pedro Vilcapaza, Arequipa, 04000, Peru; 2 Facultad de Ciencias Farmaceuticas, Bioquímicas y Biotecnológicas, Universidad Catolica de Santa Maria de Arequipa, Pedro Vilcapaza, Arequipa, 04000, Peru; 3 Sustainable Innovative Biomaterials, Le Qara Research Center, Arequipa, Peru

**Keywords:** Main protease, mutations, SARS-CoV-2, Peruvian sources, rutin

## Abstract

**Background:**

Although the COVID-19 pandemic has diminished in intensity, the virus continues to circulate globally. The SARS-CoV-2 main protease (Mpro) is a key enzyme in the life cycle of the virus, making it important for the development of treatments against future variants of the virus. In this work, Peruvian natural compounds were evaluated against different mutations of the SARS-CoV-2 Mpro.

**Methods:**

In silico techniques such as virtual screening, all-atom molecular dynamics simulations, and energy estimation analysis were applied.

**Results:**

Of the tested compounds by virtual screening, rutin was identified as the best binding agent against the different proposed Mpro mutations. In addition, computational simulations and energy estimation analysis demonstrated the high structural and energetic stability between the Mpro-rutin systems.

**Conclusions:**

Overall, our study identified rutin as the most promising compound with a strong affinity for various Mpro mutations, potentially playing a key role in the development of new treatments for emerging viral variants.

## Introduction

By the end of 2019, the world experienced the outbreak of the COVID-19 pandemic, which swiftly spread across communities and healthcare systems, causing widespread infections. ^1^ The pandemic, caused by SARS-CoV-2 (Severe Acute Respiratory Syndrome Coronavirus 2), marked a significant global health crisis. ^2^^,^^3^ Even in 2024, the effects of the virus persist, with ongoing concerns about public health and economic recovery. ^4^^,^^5^ Despite vaccination efforts and advances in treatments, COVID-19 continues to affect vulnerable populations, and the emergence of new variants remains a challenge for health systems worldwide. By late 2020, multiple variants of SARS-CoV-2 had emerged and spread rapidly, ^6^^,^^7^ and new variants have continued to evolve, further complicating global response efforts through 2024.

The SARS-CoV-2 main protease (Mpro) is a critical enzyme that plays a pivotal role in viral replication and transcription. ^8^ Upon entering the host cell, the viral RNA is translated into large polyproteins, which Mpro cleaves at specific sites to release non-structural proteins (nsps) essential for the virus’s replication. ^9^^,^^10^ Mpro specifically processes polyprotein 1ab at multiple cleavage sites and hydrolyzes the Gln-Ser peptide bond within the Leu-Gln-Ser-Ala-Gly recognition sequence. This cleavage site is unique compared to those recognized by other human cysteine proteases known to date. ^11^ As a result, Mpro has become a prime therapeutic target, with its inhibition being a promising approach to halting viral translation and replication. ^12^ Structurally, Mpro consists of three domains: domains I (residues 8-101), II (residues 102-184), and III (residues 201-306). ^8^

Likewise, several researchers have highlighted the importance of studying the stability of the Mpro structure while taking mutations into account, as this can complicate the identification of specific inhibitors. ^13^ These variants are characterized by changes in the amino acid sequence of the virus compared to the first sequenced strain, Wuhan-Hu-1 (GenBank accession: NC_045512.2). The variants may contain one or more mutations that distinguish them from the wild type. ^14^ Tracking and evaluating the spread of SARS-CoV-2 genetic variations in different countries is crucial.

It is important to note that registered mutations may alter the binding mechanisms of potential inhibitors, leading to possible resistance. ^15^ Therefore, it is essential to anticipate the effects of these mutations and identify new inhibitors to counteract them.

In the absence of a specific drug and with the emergence of new mutations, various studies are evaluating the potency of numerous phytochemicals in restricting the replication of SARS-CoV-2 and other viral infections. ^16^ Phytocompounds are considered promising drug candidates due to their high bioavailability and low toxicity. ^17^ Similarly, *in silico* studies have demonstrated the potent inhibitory action of taraxerol, found in Clerodendrum spp., a plant used in traditional medicine in tropical regions of Asia, against SARS-CoV-2 Mpro. ^18^ Additionally, *β*-amyrin and stigmasta-5,22-dien-3-ol, present in Cyperus rotundus L., a plant commonly used in traditional Indian medicine, have also shown inhibitory potential. ^19^

Peru is one of the 12 nations with the highest levels of biodiversity, which has allowed a rich tradition of medicinal practices to flourish and endure over time. ^20^ The Vavilov Institute recognizes this region as a global center for plant biodiversity. ^21^ The 20,000 to 30,000 plant species found across its diverse ecosystems account for approximately 10% of all plants used in medicine worldwide. ^22^

This study makes significant contributions to the ongoing research on novel SARS-CoV-2 Mpro inhibitors. First, it highlights natural compounds derived from Peru’s rich biodiversity, an underexplored resource in prior studies. Furthermore, we have assessed the efficacy of these compounds against eight specific Mpro mutations (Y54C, ^23^ N142S, ^23^ T190I, ^23^ A191V, ^23^ S139A, ^24^ R298A, ^24^ R60C, ^15^ and G11A ^25^), offering a comprehensive analysis of their interactions with various mutant variants of SARS-CoV-2 Mpro. Using advanced computational approaches—such as virtual screening, molecular dynamics simulations, and binding free energy estimation via the Molecular Mechanics/Generalized Born Surface Area (MM/GBSA) method—we conducted an in-depth evaluation of the structural stability and inhibitory potential of the identified compounds. Notably, our research highlights the high structural stability and potent inhibitory effects of rutin in the Mpro-rutin system. Computational simulations revealed that rutin forms stable, long-lasting interactions with the Mpro active site, underscoring its promise as a potential therapeutic candidate for COVID-19 treatment.

## Computational details

### Proteins preparation

In this study, we analyzed eight critical mutations of the SARS-CoV-2 Mpro protein, namely Y54C, N142S, T190I, A191V, S139A, G11A, R298A, and R60C. These mutations were chosen for their potential impact on the core protease’s structure and function. The Y54C mutation, identified in Malaysia, and N142S, reported in various countries, were selected because they could potentially alter the N-terminal domain’s stability and the catalytic loop’s flexibility, respectively. The T190I mutation affects the interactions within the substrate binding site, while A191V influences the dimerization dynamics, a crucial process for Mpro’s functionality. The S139A, G11A, and R298A mutations, which result in the complete loss of dimerization, are essential for Mpro’s proteolytic activity. Lastly, the R60C mutation, found in Brazil and Vietnam, affects the protein’s dynamics and the inhibitor’s ability to bind to the active site. The crystal structure of SARS-CoV-2 (PDB ID: 5RE4) reported in the Protein Data Bank ( https://www.rcsb.org/ pdb/) was used. Subsequently, mutated protein sequences were prepared by replacing the amino acids at positions R298A, N142S, A191V, R60C, G11A, Y54C, T190I, and S139A. These sequences were generated by homology modelling on the SWISS-MODEL server ( https://swissmodel.expasy.org) using the crystal structure of SARS-CoV-2 Mpro (PDB ID: 5RE4) as a template.

### Preparation of the virtual database and screening

The search for natural products was performed at the Peruvian Natural Products Database (PeruNPDB) ^26^ online web server (first version) ( https://perunpdb.com.pe/, accessed on 23 January 2022) whereas the simplified molecular-input line-entry system (SMILE) of each compound of was the upload into OpenBabel within the Python Prescription Virtual Screening Tool (PyRx) ^27^ and the subjection to energy minimization; whereas PyRx performs structure-based virtual screening by applying docking simulations using the AutoDock Vina tool. ^28^ Likewise, the FASTA sequence of the Crystal Structure of SARS-CoV-2 main protease (Mpro) (PDB: 5RE4) was subjected to a BLAST ^29^ search (accessed on 16 April 2022) whereas all the mutants were selected and subjected to automated modeling in SWISS-MODEL ^30^ server (accessed on 17 April 2022). For the analysis, the search space encompassed the whole of the modeled 3D models; and the docking simulation was then run at an exhaustiveness of eight and set to only output the lowest energy pose. Multiple sequence alignments of the Mpro and mutant sequences were visualized using the msa package (version 1.22.0) ^31^ in the R programming environment (version 4.0.3). The heatmap plot was generated using GraphPad Prism version 9.4.0 for Windows, GraphPad Software, San Diego, California USA ( www.graphpad.com).

### Molecular dynamics simulation and Molecular Mechanics/Generalized Born Surface Area (MM/GBSA) calculation

The simulation of the motion is realized by the numerical solution of the classical Newtonian dynamic equations. We used Gromacs v. 2020 ^32^ to calculate the molecular dynamics (MD) simulation and the AMBER-99SB-ILDN force field. The topologies for the Amber force field were determined on the ACPYPE server ( https://www.bio2byte.be/acpype/) for the best metabolite against mutates Mpro. Each system was included in the centre of a cube box of 10 on each side. Likewise, water molecules were added (water model TIP4P). The energy minimization was carried out with the steep-descendent integrator with 200000 calculation steps. Herein, the MD simulation in the canonical ensemble NVT was done for a time of 1ns. Finally, the production of MD continued 100 ns in the isobaric-isothermal ensemble considering the Parrinello-Rahman barostat (1 bar) and V-rescale thermostat (309.65 K). The binding free energy estimation by MM/GBSA (Molecular Mechanics/Generalized Born Surface Area) was calculated with the suit mmpbsa.py ^33^ from AmberTools20 ^34^ and gmx MMPBSA v1.4.1. ^35^ The equations related to calculations of binding free energies are the following:

$${∆G}_{\text{bind}}=G_{\text{complex}}-(G_{\text{protein}}+G_{\mathrm{lig}})$$
^(1)^

$$={\text{∆E}}_{M\;M}+{\text{∆G}}_{\mathrm{GB}}+{\text{∆G}}_{\mathrm{SA}}-T∆S$$ (2)

$$={\text{∆E}}_{\mathrm{vdw}}+{\text{∆E}}_{\mathrm{ele}}+{\text{∆G}}_{\mathrm{GB}}+{\text{∆G}}_{\mathrm{SA}}-T∆S$$ (3)

The equation that determines the electrostatic solvation energy ( *∆G _GB_*) considers ( *∆E _MM_*) which is the variation be- tween the minimized energy of the protein-ligand complexes of the study which includes the van der Waals ( *∆E _vdw_*) and electrostatic ( *∆E _ele_*) contributions, while ( *∆G _SA_*) is the difference in surface area energies for protein and ligand and ( *−T∆S*) refers to the contribution of entropy at temperature *T*.

Finally, the graphical visualizations were made with Visual Molecular Dynamics (VMD), ^36^ allowing interactive visualization with an easy-to-use interface. The interpretation of the molecular interactions was recreated with Maestro (Schrodinger) 2D interactions diagram. Likewise, the Molecular dynamics simulation results were performed by the Gromacs tools, and the values were processed by Gnuplot 5.2 ( http://gnuplot.info/) command-driven interactive function plotting program.

## Results

### Mutant SARS-CoV-2 Mpro description

SARS-CoV-2 Mpro is a cysteine protease of 67.6 kDa, and its structure possesses a catalytic dyad (Cys145 and His41) with a substrate-binding pocket located in a cleft between domains I and II. The secondary structure of Mpro has 10 alpha helixes, 13 beta sheets, 8 beta protrusions, 7 beta hairpins, 22 beta turns, 5 gamma turns, and 9 helix-helix interactions. In this work, we used the access code PDB ID: 5RE4 which was downloaded from the Protein Data Bank. This crystal structure was determined by the X-ray diffraction method with a resolution of 1.88 Å.

**Figure 1.**
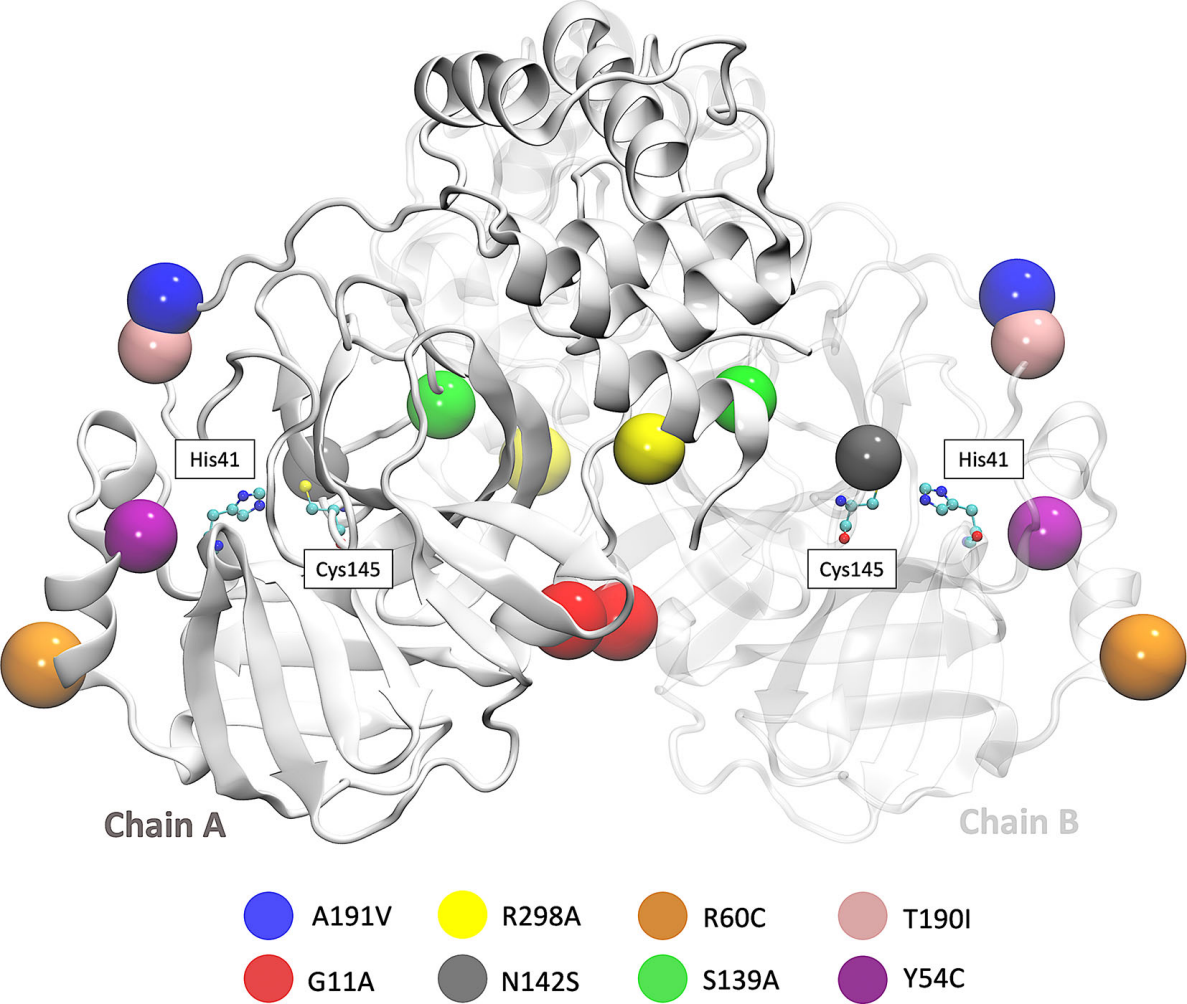
3D representation of SARS-CoV-2 Mpro in which the eight mutations are located.

Besides, we focused on the analysis of eight mutations registered in different parts of the world. First is the Y54C mutation reported in Malaysia, and the N142S mutation was reported 17 times in 5 different countries. T190I is a mutation identified in 15 countries, such as South Africa and the USA. The mutation A191V is characterized by having an occurrence rate of 0.30% and is present in more than 34 countries. Besides, the S139A, G11A, and R298A mutation results provided a better understanding of the dimerization and catalytic mechanism of the Mpro. ^24^^,^^37^ In Brazil and Vietnam, the R60C mutation was reported, affecting the protein dynamics and the inhibitor’s binding within its active site. ^15^ The R298A leads to the interruption of the dimeric conformation and irreversible inhibition of the enzyme’s catalytic activity, ^24^ and the G11A mutation avoids the insertion of the N finger region (residues 1-9) and therefore wholly declines its activity. ^38^ The location of the eight mutations is shown in Figure 1.

**Figure 2.**
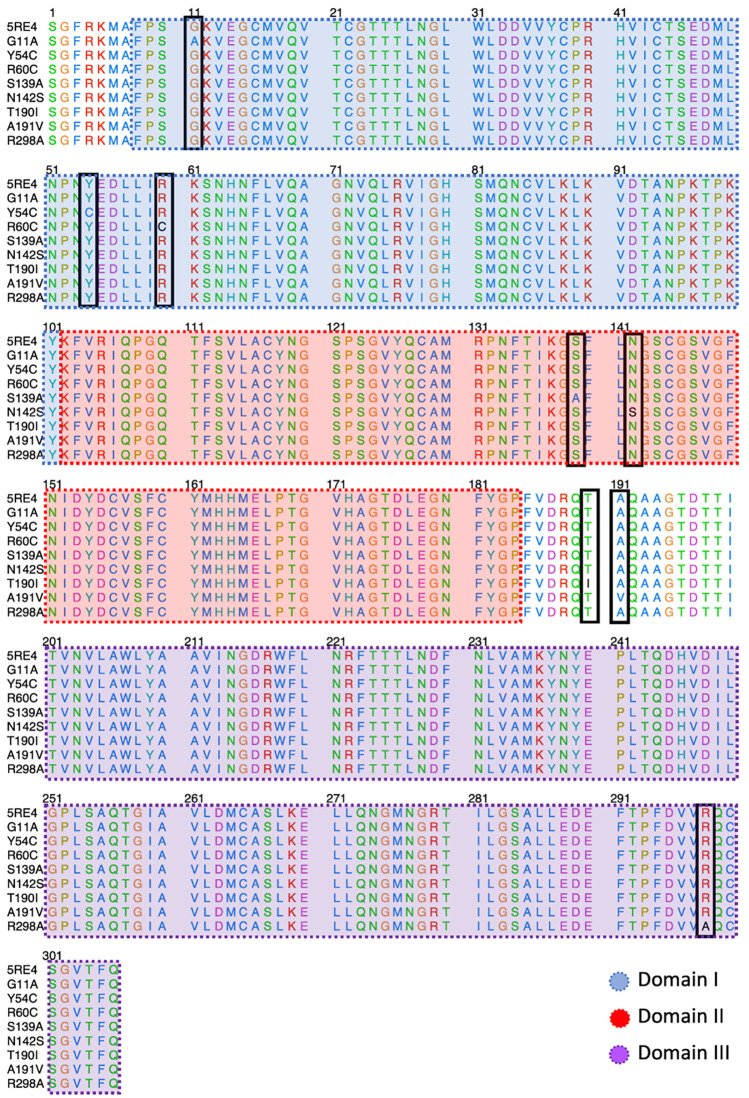
Sequence alignment of SARS-CoV-2 Mpro with the different proteins mutated.

Figure 2 shows the sequence alignment of Mpro mutations. The black square selects the variation of residues by mutant Mpro. The G11A, Y54C, and R60C mutations are located close to the His41 residue and in Domain I from Mpro. Two mutations (S139A and N142S) are present in Domain II and close to Cys145, and it is expected that these protein structures could show different behaviour than Mpro without mutations. On the other hand, it was also observed that mutations in T190I and A191V are in the connection of Domain II and Domain III. For the case of R298A mutation, it can be observed near Domain III.

The *in silico* prediction of disease-causing variants was conducted using the publicly accessible Meta-SNP tool ( https: //snps.biofold.org/meta-snp/). ^39^ This approach is distinguished by its integration of four established methods: PANTHER, PhD-SNP, SIFT, and SNAP each with predefined default threshold parameters. The prediction criteria are as follows: PANTHER, PhD-SNP, and Meta-SNP yield values between 0 and 1 (mutations with values >0.5 are predicted as disease-causing); SIFT produces a positive value (mutations with values >0.05 are predicted as neutral); and SNAP normalizes its output between 0 and 1 (mutations with values >0.5 are predicted as disease-causing). The predictions from Meta-SNP and its integrated tools provide a valuable initial assessment of the potential impact of nsSNVs (non-synonymous Single Nucleotide Variants). However, the variability in predictions underscores the importance of complementing these bioinformatic tools with experimental studies to achieve conclusive validation of each variant’s pathogenicity. Of particular interest are the Y54C and S139A mutations, which showed strong predictions towards pathogenicity and, therefore, warrant further in-depth analysis in future research (See Table 1).

**Table 1.** Summary of diseased SNPs predicted from Meta-SNP in SARS-CoV-2 mutations.

Mutation	PANTHER	PhD-SNP	SIFT	SNAP	Meta-SNP	RI	Profile
G11A	NA	Neutral	Disease	Disease	Neutral		F [G]=55%
	-	0.312	0.03	0.655	0.256	5	F [A]=0%
							Nali=46
Y54C	NA	Neutral	Disease	Disease	Disease		F [Y]=64%
	-	0.499	0	0.745	0.675	4	F [C]=0%
							Nali=46
R60C	NA	Neutral	Neutral	Disease	Neutral		F [R]=56%
	-	0.435	0.16	0.630	0.323	4	F [C]=0%
							Nali=47
S139A	NA	Disease	Disease	Disease	Disease		F [S]=100%
	-	0.535	0	0.79	0.73	5	F [A]=0%
							Nali=47
N142S	NA	Neutral	Neutral	Neutral	Neutral		F [N]=33%
	-	0.189	0.7	0.47	0.193	6	F [S]=4%
							Nali=47
T190I	NA	Neutral	Neutral	Neutral	Neutral		F [T]=17%
	-	0.214	0.34	0.39	0.197	6	F [I]=4%
							Nali=46
A191V	NA	Neutral	Neutral	Neutral	Neutral		F [A]=32%
	-	0.066	1	0.3	0.064	9	F [V]=26%
							Nali=46
R298A	NA	Neutral	Neutral	Disease	Neutral		F [R]=30%
	-	0.159	0.06	0.53	0.183	6	F [A]=0%
							Nali=46

RI=Reliability Index between 0 and 10; F [X]=Frequency of residue X in the sequence profile; Nali: Number of aligned sequences in the mutated site.

### Virtual screening analysis

The Virtual Screening technique, widely used for drug discovery, seeks to identify potential compounds for a particular therapeutic target. This approach allowed us to find new possible candidates within the PeruNPDB dataset against one of the therapeutic targets from mutant Mpro of SARS-CoV-2.

**Figure 3.**
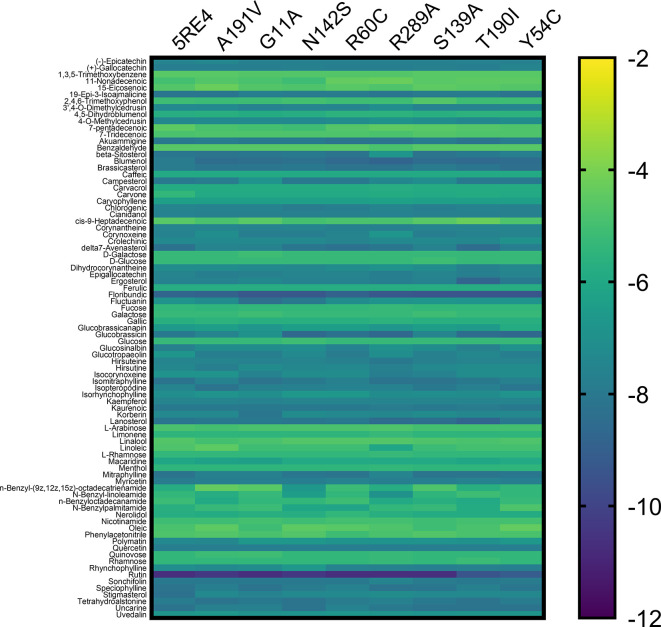
Heat map analysis of binding constants of metabolites from Peruvian native plants screened against mutated Mpro of SARS-CoV-2.

The 84 substances included in the study are taken from the original PeruNPDB collection; the most recent dataset consists of 280 substances. Figure 3 shows the gradient palette, the violet color indicated strong binding ( *∆*G<-12 kcal/mol), while the yellow color indicated weak binding ( *∆*G>-2 kcal/mol). In this heat map, rutin is shown as the best compounds. However, for the T190I and Y54C mutations, the color intensity is lower compared to the other mutations.

In Table S1 of the Supplementary Material, the values of coupling energies are reported. The values for Mpro wild, A191V, G11A, N142S, R60C, R289C, S139A, T190I, and Y54C were -10.7 kcal/mol, -10.4 kcal/mol, -10.7 kcal/mol, -10.4 kcal/mol, - 10.7 kcal/mol, -10.7 kcal/mol, -10.7 kcal/mol, -9.4 kcal/mol, and -9.1 kcal/mol, respectively. Additionally, the results of Lipinski’s rule of five ^40^ and ADMET (Absorption, Distribution, Metabolism, Excretion and Toxicity) prediction obtained from http://www.scfbio-iitd.res.in/ ADMETlab v 3.0 ^41^ of the 84 compounds are shown in Table S2 and Table S3 from the Supplementary Material. The Lipinski’s rule of five analysis for the majority of the compounds revealed no significant violations in terms of molecular weight, hydrogen bond donors, and acceptors. However, some compounds did not comply with the logP values. Further experimental determination of logP values may provide a better understanding of these discrepancies. The data obtained from the ADMET analysis were found to be comprehensive and informative, allowing for a better understanding of the properties to be considered in the present study.

### Molecular dynamics simulations and estimation of binding free energy

**Figure 4.**
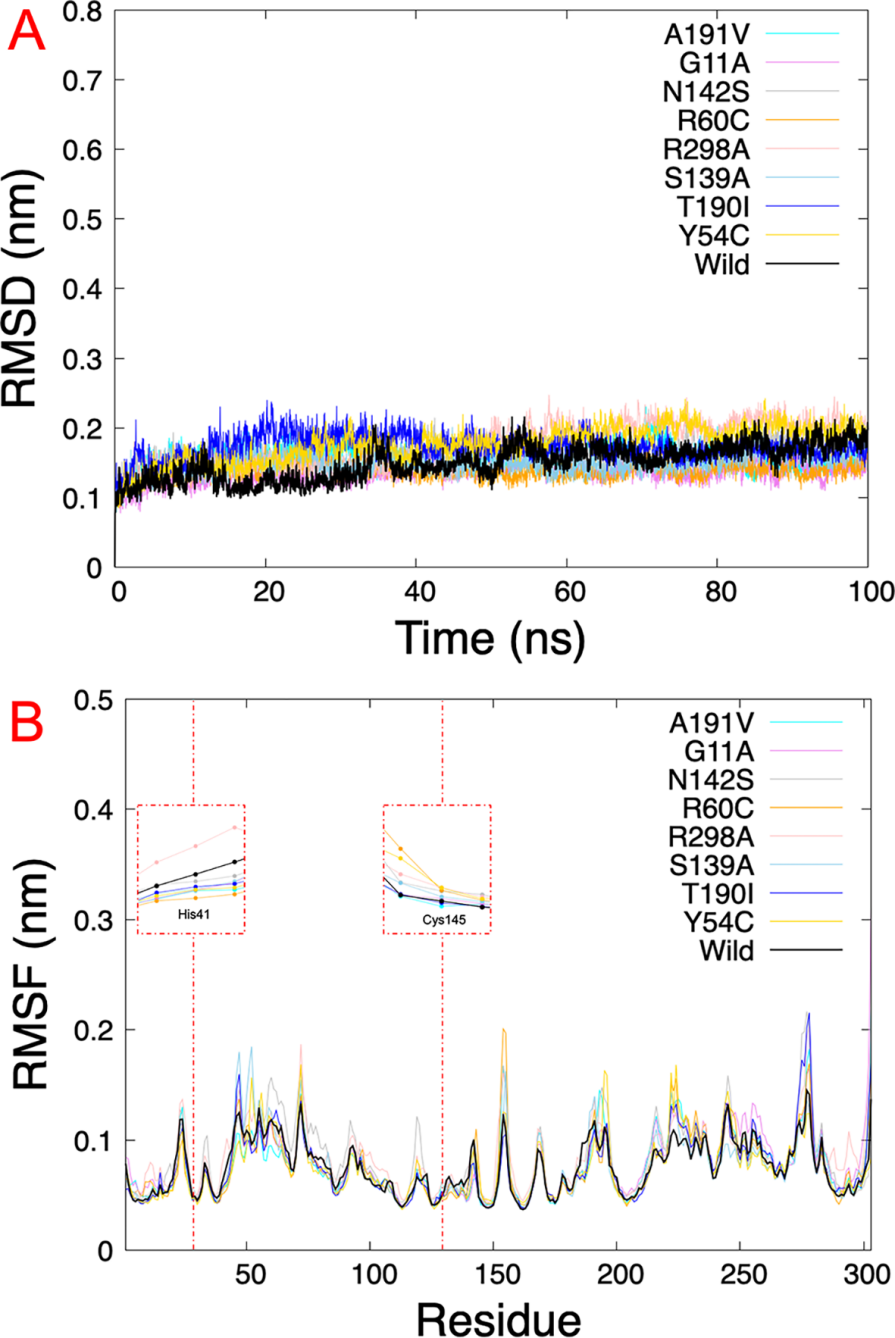
RMSD and RMSF plots. A.) RMSD of eight SARS-CoV-2 Mpro with rutin. B.) RMSF of the last 5 ns *per residue* of each SARS-CoV-2 Mpro mutated, highlighting the principal residues of the catalytic dyad (His41 and Cys145). RMSD, Root-mean square deviation; RMSF, Root-mean square fluctuation; SARS-CoV-2, severe acute respiratory syndrome coronavirus 2; Mpro, main protease.

The results obtained from virtual screening helped us to consider rutin as a ligand against the different Mpro mutations. Molecular dynamics simulations allow us to understand the behavior of different mutated Mpro at an atomistic level. After analyzing 100 ns of production dynamics, the convergence of each protein is observed by Root-mean squared deviation (RMSD) analysis (See Figure 2A). This result shows us that the different types of mutations achieved equilibrium; likewise, an average RMSD between 0.1 and 0.2 nm is appreciated, an acceptable value in this structural model. The Root-mean squared fluctuation (RMSF) calculates the flexibility of individual residues that make up the Mpro protein during a simulation trajectory. The RMSF *per residue* diagram structurally indicates which amino acids in a protein contribute the most to a molecular motion. Figure 4B highlights the area of His41 and Cys145 amino acids where the most significant fluctuation in the His41 area occurs with the R298A mutation, while the most significant fluctuation in the Cys145 area occurred in the R60C, Y54C, R298A, and N142S mutation.

On the other hand, Table 2 shows us the quantitative values of the RMSD, where the G11A and R60C mutations showed the lowest average RMSD value. In contrast, the average value for the Y54C mutation was higher than the others. Regarding the average RMSF values of the last 5 ns, for the 5RE4, A191V, G11A, N142S, R60C, S139A, and T190I systems, the average RMSF of the Mpro structures oscillated by 0.8 nm, while for R298A the RMSF average results in 0.09 nm and Y54C it was 0.07 nm.

**Table 2.** RMSD and RMSF average values of SARS-CoV-2 Mpro wild and mutated.

System	RMSD (nm)	RMSF (nm)
5RE4	0.15 *±*0.02	0.08 *±*0.03
A191V	0.16 *±*0.02	0.08 *±*0.03
G11A	0.14 *±*0.02	0.08 *±*0.04
N142S	0.16 *±*0.02	0.08 *±*0.03
R60C	0.14 *±*0.01	0.08 *±*0.03
R298A	0.17 *±*0.03	0.09 *±*0.03
S139A	0.15 *±*0.02	0.08 *±*0.03
T190I	0.17 *±*0.02	0.08 *±*0.03
Y54C	0.18 *±*0.03	0.07 *±*0.03

Besides virtual screening and molecular dynamics simulation studies, molecular mechanics/generalized Born surface area (MM/GBSA) was performed with all frames of the MD. Table 3 indicates the average free energy values for each system. The values show a high coupling energy estimate, indicating that the interaction was carried out correctly.

The mutation R60C showed the best interaction energy (-45.09 kcal/mol) against the different systems studied. The energy values for G11A and A191V were -41.17 kcal/mol and -40.71 kcal/mol, respectively. While the systems that showed low binding energy were mutations R298A and S139A, with average values of -24.11 kcal/mol and -25.84 kcal/mol, respectively.

**Table 3.** Calculated Molecular Mechanics/Generalized Born Surface Area (MM/GBSA) binding free energy of the systems.

System	∆ TOTAL	VDWAALS	EEL	EGB	∆G gas	∆G solv
5RE4	-33.49±7.32	-44.09±6.48	-33.36±16.91	50.02±9.38	-77.45±14.88	43.96±9.18
A191V	-40.71±6.07	-47.19±4.43	-43.39±10.75	55.74±7.21	-90.58±12.04	49.88±7.03
G11A	-41.17±4.48	-46.51±3.29	-42.57±10.84	53.40±7.96	-89.08±10.55	47.91±7.78
N142S	-36.65±3.82	-54.18±3.16	-26.84±6.44	51.02±5.15	-81.02±6.77	44.37±5.10
R60C	-45.09±7.29	-48.90±5.88	-49.45±12.23	59.24±7.93	-98.35±13.21	53.26±7.10
R298A	-24.11±8.41	-36.30±8.91	-21.09±12.85	37.81±12.91	-57.39±19.44	33.28±11.74
S139A	-25.84±2.95	-45.56±2.63	-18.41±7.38	43.84±5.14	-63.97±6.74	38.13±5.11
T190I	-35.87±5.80	-48.17±5.82	-30.99±8.97	49.32±5.99	-79.15±9.71	43.29±5.81
Y54C	-34.53±5.62	-28.04±5.52	-66.78±10.26	65.39±6.59	-94.82±10.55	60.29±6.36

Likewise, the most significant energy contribution was given by the Van der Waals energies ( *∆*E *_V DWAALS_*) in the wild system, A191V, G11A, N142S, R298A, S139A, and T190I. These types of energy are weak and have short-range inter- actions; in biological systems, they play a significant role in stabilizing protein-small molecules. On the other hand, in the R60C and Y54C systems, the energy contribution is given by electrostatic energies ( *∆*E *_ELE_*). Electrostatic energy considers into account the charges of each atom in the system, which depend on the medium in which they are found; these have greater scope, and the force of interaction it possesses is linked to the relative orientations it accepts.

**Figure 5.**
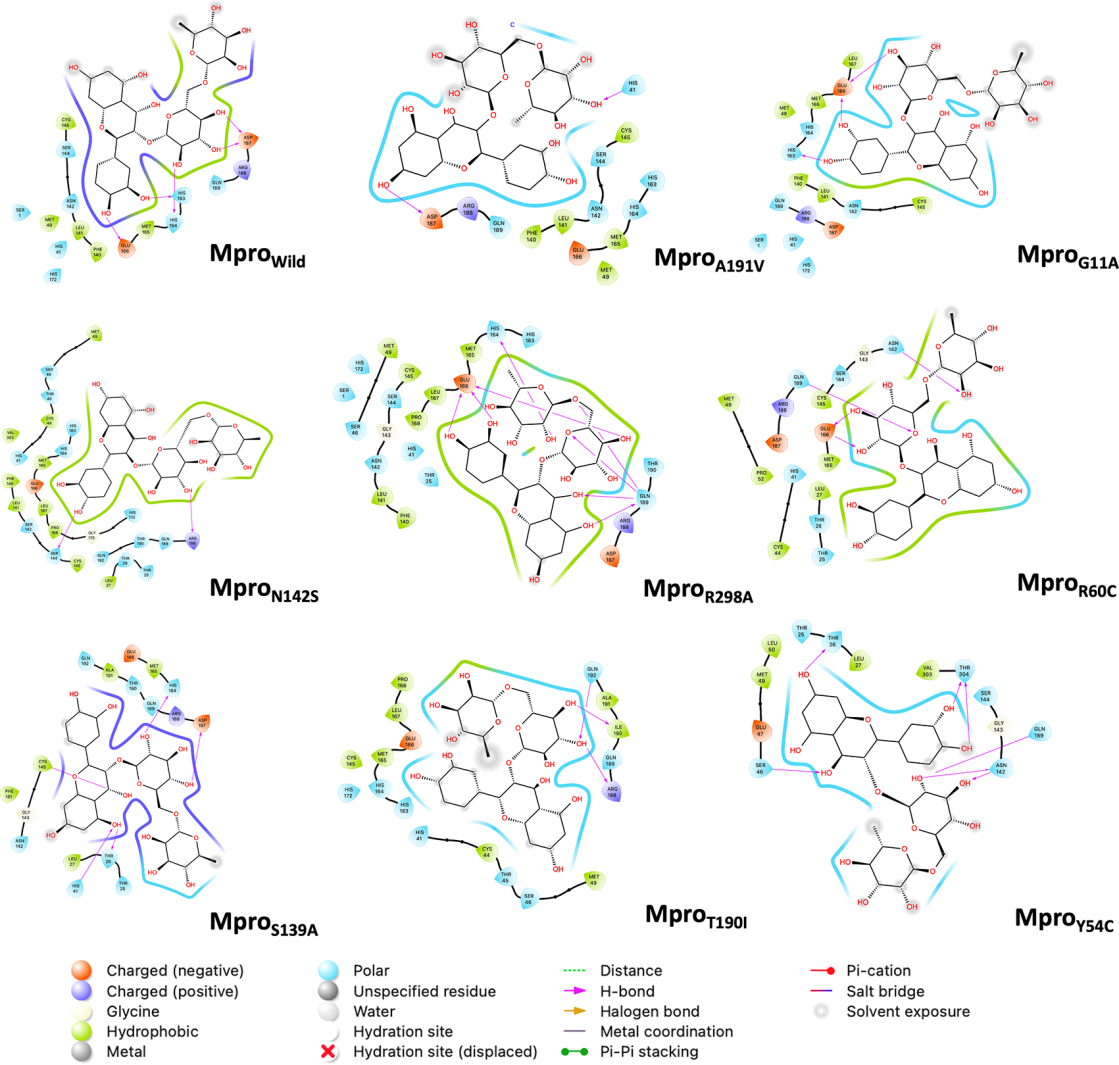
2D interaction diagram of rutin. The pink arrow lines represent the hydrogen bond. Mpro, main protease.

Figure 5 shows the last frame of each simulation of the Mpro-rutin complex. In general, it was observed that the interactions in the active site are due to the formation of hydrogen bonds. However, we observed some changes in the region around the active site for the mutations occurring in N142S and Y54C. The residues around N142S are mostly hydrophobic (green contour), hence N142S exhibits a greater energy contribution from hydrophobic interactions ( *∆*E *_V DWAALS_* = -54.18 kcal/mol, higher than the other mutations). In Y54C, the residues around rutin are polar (sky blue contour), demonstrating its high energetic contribution by electrostatic interactions ( *∆*E *_ELE_* = -66.78 kcal/mol more elevated than the other mutations).

## Discussion

To date, research teams worldwide have been collecting data on SARS-CoV-2 strains, some of which exhibit numerous mutations in various structural proteins. The main protease (Mpro) plays a crucial role in the SARS-CoV-2 life cycle by mediating viral replication and transcription. Mpro functions by cleaving the viral polyproteins pp1a and pp1ab, which are synthesized from the viral RNA once SARS-CoV-2 enters the host cell. It recognizes specific sequences within these polyproteins and cleaves them at approximately 11 conserved sites, releasing nonstructural proteins (nsps) essential for forming the replication-transcription complex (RTC). The active site of Mpro, consisting of cysteine and histidine, catalyzes the cleavage of peptide bonds, thereby enabling the release of nsps required for viral replication. This makes Mpro a key target for therapeutic interventions.

However, mutations in the main protease (Mpro) of SARS-CoV-2 are significant because they can influence the virus’s ability to replicate and its susceptibility to antiviral treatments. Mpro is a critical enzyme in processing viral polyproteins, and any alterations in its structure due to mutations can affect its catalytic efficiency, protein stability, and interaction with inhibitors. ^42^

In this context, numerous studies have focused on identifying new inhibitors derived from natural compounds, as they offer a rich and promising source for drug discovery against SARS-CoV-2. ^43– 52^ These compounds provide significant advantages in terms of chemical diversity, safety, sustainability, and therapeutic efficacy. For example, several phytochemical molecules, such as kaempferol, quercetin, luteolin-7-glucoside, demethoxycurcumin, naringenin, apigenin-7-glucoside, oleuropein, curcumin, catechin, and epicatechin gallate, have been reported as promising antiviral agents against SARS-CoV-2. ^53^ Additionally, Parvez et al. identified azobechalcone, rifampin, isolophirachalcone, tetrandrine, and fangchinoline as potential Mpro inhibitors, ^54^ while Padhi et al. demonstrated that putaminoxin B, putaminoxin D, jasmonic acid, and jasmonic methyl ester possess good pharmacokinetic properties against Mpro. ^55^ In 2020, more than a thousand FDA-approved drugs were virtually screened using molecular docking and binding free energy calculations, with nelfinavir emerging as a potential inhibitor of SARS-CoV-2. Goyzueta et al. studied rutin as a promising Mpro inhibitor using *in silico* techniques. ^56^ Similarly, reused drugs and phytochemical compounds have shown binding affinities to various Mpro mutants. For instance, salvianolic acid A, extracted from *Salvia miltiorrhiza*, demonstrated inhibition effects against the N142S and T190I mutations. ^23^

We analyzed eight specific Mpro mutations (Y54C, N142S, T190I, A191V, S139A, G11A, R298A, and R60C), which have been identified in various parts of the world and have significant implications for the protease’s structure and function. These mutations are critical for developing effective inhibitors for antiviral treatments. The Y54C mutation, found in Malaysia, may affect the stability of Mpro, potentially altering its overall structure and interaction with inhibitors. ^57^ This could compromise the three-dimensional conformation of Mpro, which is essential for its active site function. The N142S mutation, reported in five countries, could impact the flexibility of the catalytic loop, a key element in the active site’s efficiency. ^58^ Alterations in this region may significantly affect the protease’s functionality. The T190I mutation, identified in 15 countries, including South Africa and the United States, may alter interactions at the substrate binding site by changing the orientation of catalytic residues, which could impair Mpro’s ability to interact with substrates. ^59^^,^^60^ The A191V mutation, with a 0.30% occurrence rate, has been reported in over 34 countries and may influence dimerization dynamics, which are essential for enzymatic function, thereby affecting the stability and activity of Mpro. ^38^^,^^61^ The S139A mutation could modify the catalytic environment, affecting Mpro’s interaction with inhibitors. ^62^ The R298A mutation disrupts dimeric conformation and leads to the irreversible inhibition of catalytic activity, destabilizing Mpro’s structure and impairing its ability to maintain an active conformation. ^24^ The G11A mutation eliminates the N-finger region (residues 1-9), reducing Mpro’s enzymatic activity and preventing proper dimer formation, which is crucial for proteolytic function. ^38^ The R60C mutation, identified in Brazil and Vietnam, affects the protein’s dynamics and the inhibitor binding within its active site, thus compromising Mpro inhibition. ^15^ This mutation particularly impacts Mpro’s three-dimensional structure and its interaction with potential therapeutic inhibitors, thereby reducing the effectiveness of protease inhibitor-based treatments.

The novelty of this study lies in the use of 84 substances taken from the original PeruNPDB ( https://perunpdb.com.pe) collection; the most recent dataset consists of 280 substances. Our results reveal that the rutin metabolite, found in *Smallanthus sonchifolius* (yacón) and *Lepidium meyenii* (maca), exhibited the strongest binding affinity with all the proposed Mpro mutations. Rutin, also known as rutoside, is a natural phenolic compound that plays a key role in maintaining the oxidant-antioxidant balance associated with certain diseases. ^63^^,^^64^ The MD simulation results indicate that rutin interacted with the active site residues of Mpro mutations and remained stabilized in the active site region with minimal fluctuation. These results are in perfect agreement with the MM/GBSA obtained after the docking calculation and the RMSD analysis. Where the stable RMSD ensures that the MM/GBSA calculation is based on a structurally consistent system, making the free energy predictions more reliable between Mpro mutations and rutin.

## Conclusions

The main protease (Mpro) is crucial for SARS-CoV-2 replication and represents a promising drug target. In this study, we analyzed eight Mpro mutations of SARS-CoV-2 (Y54C, N142S, T190I, A191V, S139A, R298A, R60C, and G11A), each located in different regions of the protease. Among these, S139A demonstrated strong pathogenicity predictions through Meta-SNP calculations, warranting further investigation in future research. Additionally, virtual screening identified rutin, from the PeruNPDB database, as the most promising candidate for binding to Mpro. Molecular dynamics simulations and energy estimation analyses confirmed that rutin forms a highly stable complex with Mpro. We believe that computer-assisted drug design and molecular dynamics simulations offer a powerful complementary approach to screening potential Mpro inhibitors, providing an attractive strategy to combat SARS-CoV-2 and its variants.

### Ethics and consent

Ethical approval and consent were not required.

## Author contributions

Conceptualization: H.L.B.-C. and L.D.G.-M.; data curation: H.L.B.-C., L.D.G.-M., E.G.C.-R., A.E.C.-L., M.P.-C., and M.A.C.-F; formal analysis: H.L.B.-C. and M.A.C.-F.; funding acquisition: H.L.B.-C. and M.A.C.-F.; investigation: H.L.B.-C., M.P.-C., L.D.G.-M., E.G.C.-R., A.E.C.-L., and M.A.C.-F; methodology: H.L.B.-C. and M.A.C.-F.; writing—review and editing: H.L.B.-C., and M.A.C.-F. All authors have read and agreed to the published version of the manuscript.

## Data availability

### Underlying data

Figshare: Dataset of results from virtual screening and molecular dynamics simulations. https://doi.org/10.6084/m9.figshare.27156276.v1. ^65^

This project contains the following underlying data:

• Table 1. Summary of diseased SNPs predicted from Meta-SNP in SARS-CoV-2 mutations.
• Table 2. RMSD and RMSF average values of SARS-CoV-2 Mpro wild and mutated.
• Table 3. Calculated MM/GBSA binding free energy of the systems.
• Table S1. Values of the coupling energies obtained by virtual screening.
• Table S2. Lipinski’s “rule of five” analysis of the 84 phytocompounds determined their solubility, permeability, and efficacy for drug discovery.
• Table S3. In silico ADMET properties of 84 phytocompounds.

Data are available under the terms of the Creative Commons Attribution 4.0 International license (CC-BY 4.0).
