# Supplementary material for: Identification of compounds from natural Peruvian sources as potential inhibitors of SARS-CoV-2 Mpro mutations by virtual screening and computational simulations
Source: F1000Res. 2024 Nov 22;13:246. Originally published 2024 Apr 4. [Version 3] doi: 10.12688/f1000research.143633.3 (PMC11585855; doi:10.12688/f1000research.143633.3)
Supplement: Peer review history

# Reviewer report: Reviewer response for version 3

**DOI:** 10.5256/f1000research.175019.r342657

**Published:** 2024-11-23

**Authors:** Tushar Joshi

**Affiliations:** 1 Biotechnology, Vellore Institue of technology, Vellore, Tamil Nadu, India

The authors have addressed all the reviewers comments. I hereby recommend its acceptance.

Is the work clearly and accurately presented and does it cite the current literature?

Partly

If applicable, is the statistical analysis and its interpretation appropriate?

Not applicable

Are all the source data underlying the results available to ensure full reproducibility?

Yes

Is the study design appropriate and is the work technically sound?

Yes

Are the conclusions drawn adequately supported by the results?

Partly

Are sufficient details of methods and analysis provided to allow replication by others?

Partly

Reviewer Expertise:

Antibacterial and Antiviral Resistance

I confirm that I have read this submission and believe that I have an appropriate level of expertise to confirm that it is of an acceptable scientific standard.

# Reviewer report: Reviewer response for version 2

**DOI:** 10.5256/f1000research.172930.r336155

**Published:** 2024-11-22

**Authors:** Mohammed Bouachrine

**Affiliations:** 1 Department of Chemistry, University of Moulay Ismail, Meknes, Morocco

Dear editor

 After a detailed reading of the manuscript, I noticed that the authors respected and responded favorably to my comments and questions. The results are interesting and I recommend its indexing in this form.

Is the work clearly and accurately presented and does it cite the current literature?

Yes

If applicable, is the statistical analysis and its interpretation appropriate?

Partly

Are all the source data underlying the results available to ensure full reproducibility?

Yes

Is the study design appropriate and is the work technically sound?

Yes

Are the conclusions drawn adequately supported by the results?

Partly

Are sufficient details of methods and analysis provided to allow replication by others?

Yes

Reviewer Expertise:

Drug Design, Molecular Modeling, in-silico studies

I confirm that I have read this submission and believe that I have an appropriate level of expertise to confirm that it is of an acceptable scientific standard.

# Reviewer report: Reviewer response for version 2

**DOI:** 10.5256/f1000research.172930.r336157

**Published:** 2024-11-06

**Authors:** Tushar Joshi

**Affiliations:** 1 Biotechnology, Vellore Institue of technology, Vellore, Tamil Nadu, India

The submitted revisions have significantly improved the quality of the manuscripts. The authors have addressed all reviewer comments, but they have not cited the articles mentioned in the comments. Authors are suggested to cite the articles in the appropriate place. I recommend accepting it after this minor correction.

Is the work clearly and accurately presented and does it cite the current literature?

Partly

If applicable, is the statistical analysis and its interpretation appropriate?

Not applicable

Are all the source data underlying the results available to ensure full reproducibility?

Yes

Is the study design appropriate and is the work technically sound?

Yes

Are the conclusions drawn adequately supported by the results?

Partly

Are sufficient details of methods and analysis provided to allow replication by others?

Partly

Reviewer Expertise:

Antibacterial and Antiviral Resistance

I confirm that I have read this submission and believe that I have an appropriate level of expertise to confirm that it is of an acceptable scientific standard, however I have significant reservations, as outlined above.

# Response

**Published:** 2024-11-19

**Authors:** Miguel Angel Chavez-Fumagalli

**Affiliations:** Universidad Catolica de Santa Maria, Peru

We thank the reviewer for his contribution to improving the manuscript. Accordingly, we have added the references to the newest version of the paper.

# Reviewer report: Reviewer response for version 1

**DOI:** 10.5256/f1000research.157315.r290175

**Published:** 2024-06-25

**Authors:** Mohammed Bouachrine

**Affiliations:** 1 Department of Chemistry, University of Moulay Ismail, Meknes, Morocco

Today, numerous studies are studying the different mutations of SARS-CoV-2 which are worrying and which can make the virus more aggressive and spread faster. Along these lines, the authors studied eight SARS-CoV-2 Mpro mutations (Y54C, N142S, T190I, A191V, S139A, R298A, R60C, and G11A) and analyzed several compounds from Peruvian natural sources by virtual screening methods. Molecular dynamics simulations and binding free energy estimation by MM/GBSA showed high stability of the Mpro-rutin complex and excellent energy affinity, respectively. These results demonstrated the database the utility of PeruNPDB in finding rutin as a promising inhibitor of different SARS-CoV-2 Mpro mutations.

 The results are numerous and interesting, the article corresponds perfectly to the objectives of the journal. I recommend its publication after a few corrections

  

1. Authors are invited to better rewrite the abstract and the conclusion
2. The authors are invited to explain and to detail the mechanism of action
3. The authors must justify the choice of the protein
4. Why did the authors not pursue the studies using other in-silico techniques
5. Authors must cite work published in this journal and other journals on the same studies

 

- **Acta Pharm. Sin. B (2020),** **10.1016/j.apsb.2020.02.008
**
- **
https://doi.org/10.1016/j.molstruc.2022.132652
**
- **
https://doi.org/10.1080/07391102.2020.1758790
**
- **Life Sci., 251 (2020), Article 117627, 10.1016/j.lfs.2020.117627
**
- **
https://doi.org/10.2174/1386207323999200730205447
**
- **
https://doi.org/10.1038/nature17180, 2-s2.0-84982234143.**
- **
https://doi.org/10.1080/07391102.2020.1779130
**
- **
https://doi.org/10.1016/j.compbiomed.2021.104758
**
- **
https://doi.org/10.1155/2021/1901484
**

Is the work clearly and accurately presented and does it cite the current literature?

Yes

If applicable, is the statistical analysis and its interpretation appropriate?

Partly

Are all the source data underlying the results available to ensure full reproducibility?

Yes

Is the study design appropriate and is the work technically sound?

Yes

Are the conclusions drawn adequately supported by the results?

Partly

Are sufficient details of methods and analysis provided to allow replication by others?

Yes

Reviewer Expertise:

Drug Design, Molecular Modeling, in-silico studies

I confirm that I have read this submission and believe that I have an appropriate level of expertise to confirm that it is of an acceptable scientific standard, however I have significant reservations, as outlined above.

## References

[1] : Analysis of therapeutic targets for SARS-CoV-2 and discovery of potential drugs by computational methods. *Acta Pharm Sin B* .2020;10(5) : 10.1016/j.apsb.2020.02.008 766-788 10.1016/j.apsb.2020.02.008 32292689 PMC7102550

[2] : In silico detection of potential inhibitors from vitamins and their derivatives compounds against SARS-CoV-2 main protease by using molecular docking, molecular dynamic simulation and ADMET profiling. *J Mol Struct* .2022;1258: 10.1016/j.molstruc.2022.132652 132652 10.1016/j.molstruc.2022.132652 35194243 PMC8855669

[3] : Moroccan Medicinal plants as inhibitors against SARS-CoV-2 main protease: Computational investigations. *J Biomol Struct Dyn* .2021;39(8) : 10.1080/07391102.2020.1758790 2971-2979 10.1080/07391102.2020.1758790 32306860 PMC7212546

[4] : Virtual screening and repurposing of FDA approved drugs against COVID-19 main protease. *Life Sci* .2020;251: 10.1016/j.lfs.2020.117627 117627 10.1016/j.lfs.2020.117627 32251634 PMC7194560

[5] : Discovery of Potent SARS-CoV-2 Inhibitors from Approved Antiviral Drugs via Docking and Virtual Screening. *Comb Chem High Throughput Screen* .2021;24(3) : 10.2174/1386207323999200730205447 441-454 10.2174/1386207323999200730205447 32748740

[6] : Therapeutic efficacy of the small molecule GS-5734 against Ebola virus in rhesus monkeys. *Nature* .2016;531(7594) : 10.1038/nature17180 381-5 10.1038/nature17180 26934220 PMC5551389

[7] : Identification of a novel dual-target scaffold for 3CLpro and RdRp proteins of SARS-CoV-2 using 3D-similarity search, molecular docking, molecular dynamics and ADMET evaluation. *J Biomol Struct Dyn* .2021;39(12) : 10.1080/07391102.2020.1779130 4522-4535 10.1080/07391102.2020.1779130 32552534 PMC7309310

[8] : Camphor, Artemisinin and Sumac Phytochemicals as inhibitors against COVID-19: Computational approach. *Comput Biol Med* .2021;136: 10.1016/j.compbiomed.2021.104758 104758 10.1016/j.compbiomed.2021.104758 34411900 PMC8354799

[9] : Identification of Novel SARS-CoV-2 Inhibitors: A Structure-Based Virtual Screening Approach. *Journal of Chemistry* .2021;2021: 10.1155/2021/1901484 1-7 10.1155/2021/1901484

# Response

**Published:** 2024-10-03

**Authors:** Miguel Angel Chavez-Fumagalli

**Affiliations:** Universidad Catolica de Santa Maria, Peru

Today, numerous studies are studying the different mutations of SARS-CoV-2 which are worrying and which can make the virus more aggressive and spread faster. Along these lines, the authors studied eight SARS-CoV-2 Mpro mutations (Y54C, N142S, T190I, A191V, S139A, R298A, R60C, and G11A) and analyzed several compounds from Peruvian natural sources by virtual screening methods. Molecular dynamics simulations and binding free energy estimation by MM/GBSA showed high stability of the Mpro-rutin complex and excellent energy affinity, respectively. These results demonstrated the database the utility of PeruNPDB in finding rutin as a promising inhibitor of different SARS-CoV-2 Mpro mutations.

 The results are numerous and interesting, the article corresponds perfectly to the objectives of the journal. I recommend its publication after a few corrections

 Authors are invited to better rewrite the abstract and the conclusion

 Answer: The abstract and conclusions were improved

 The authors are invited to explain and to detail the mechanism of action

 Answer: The mechanism of action of Mpro in SARS-CoV-2 is explained in the “Introduction” section.

 The authors must justify the choice of the protein

 Answer: It was better explained in the “Introduction” section

 Why did the authors not pursue the studies using other in-silico techniques

 Answer: Our expertise lies in the employment of in-silico methodologies that are both reliable and widely recognized within the scientific community for the investigation of viral proteins. The decision to abstain from utilizing additional in-silico techniques was based on practical and strategic considerations. Initially, the techniques implemented in this study, including virtual screening, molecular dynamics simulations, and binding free energy estimation using the Molecular Mechanics/Generalized Born Surface (MM/GBSA) method, have already demonstrated exceptional effectiveness and dependability in the structural and functional analysis of viral proteins. These techniques provided an in-depth understanding of the molecular interactions between Mpro and the chosen natural compounds. It should be noted that the inclusion of more in-silico techniques could have increased the complexity and duration of the study without guaranteeing a proportional improvement in the quality of the results. It is important to emphasize that virtual screening was utilized to efficiently identify potentially effective compounds from a vast library of molecules, while molecular dynamics simulations were essential to examine atomic-level interactions between inhibitors and Mpro in a dynamic environment. These simulations provided detailed information on the structural stability of the complexes formed and the possible conformations adopted during the interaction. The MM/GBSA method allowed for the quantification of the affinity of the inhibitors for Mpro. Our primary objective was to provide precise and reproducible data within a reasonable time frame. By concentrating on well-established methodologies, we ensure the consistency and validity of our results, avoiding potential complications and variations associated with the use of lesser-known or invalidated techniques in the context of this specific study.

 Authors must cite work published in this journal and other journals on the same studies: Acta Pharm. Sin. B (2020), 10.1016/j.apsb.2020.02.008

https://doi.org/10.1016/j.molstruc.2022.132652

https://doi.org/10.1080/07391102.2020.1758790

10.1016/j.lfs.2020.117627

https://doi.org/10.2174/1386207323999200730205447

https://doi.org/10.1038/nature17180, 2-s2.0-84982234143.

https://doi.org/10.1080/07391102.2020.1779130

https://doi.org/10.1016/j.compbiomed.2021.104758

https://doi.org/10.1155/2021/1901484

 Answer: Citations have been incorporated

# Reviewer report: Reviewer response for version 1

**DOI:** 10.5256/f1000research.157315.r269268

**Published:** 2024-05-17

**Authors:** Tushar Joshi

**Affiliations:** 1 Biotechnology, Vellore Institue of technology, Vellore, Tamil Nadu, India

**Editorial note from F1000Research: This report was originally published on 17th May 2024 with an approval status of 'Approved', but the reviewer has now clarified that their approval status was intended to be 'Approved with reservations', so on 27th May 2024 the approval status of the report has been updated accordingly to reflect this.**

 The research article entitled “Identification of compounds from natural Peruvian sources as potential inhibitors of SARS-CoV-2 Mpro mutations by virtual screening and computational simulations” has found rutin is a potential inhibitor against SARS-CoV-2 Mpro mutations. The research topic is interesting. There are recommendations for the authors to make the manuscript more effective for readers. The manuscript can be considered for indexing on addressing the following major comments.

1. The abstract conclusion is not that effective, it could be more effective. It should not start with “his”, kindly rephrase it.
2. The abstract is not very effective, kindly re-write it. Kindly refer to these research papers for better understanding: Mathpal *et al.* (2022( ^1^) and Joshi *et al. *(2022 ^2^) and cite them accordingly.
3. The authors have not analyzed the properties of mutations either these are deleterious in nature or neutral mutation. There are many online servers like Meta-SNP. Authors can use these servers.
4. The authors have not analyzed the ADMET and Lipinski properties of compounds. Kindly include these methods.
5. In the results, the authors have written that mutation is collected from different sources. The short part should be written on the material method and a long can be written on the discussion part.
6. The results of RMSD are good but for better understanding kindly analyze interaction energy.
7. The authors have been unable to explain how this study is different from other studies that have also found rutin against Mpro.
8. There are many loopholes in this study. 
9. The discussion and conclusion are also not written perfectly. Kindly try to cite recent publications.
10. There is a high recommendation for authors to use professional software or language experts to correct the grammar and language of the manuscript. There are many places where sentences look incomplete and create confusion for readers. 

Is the work clearly and accurately presented and does it cite the current literature?

Partly

If applicable, is the statistical analysis and its interpretation appropriate?

Not applicable

Are all the source data underlying the results available to ensure full reproducibility?

Yes

Is the study design appropriate and is the work technically sound?

Yes

Are the conclusions drawn adequately supported by the results?

Partly

Are sufficient details of methods and analysis provided to allow replication by others?

Partly

Reviewer Expertise:

Antibacterial and Antiviral Resistance

I confirm that I have read this submission and believe that I have an appropriate level of expertise to confirm that it is of an acceptable scientific standard, however I have significant reservations, as outlined above.

## References

[1] : Assessment of activity of chalcone compounds as inhibitors of 3-chymotrypsin like protease (3CLPro) of SARS-CoV-2: in silico study. *Struct Chem* .2022;33(5) : 10.1007/s11224-022-01887-2 1815-1831 10.1007/s11224-022-01887-2 35153452 PMC8818839

[2] : Computational investigation of drug bank compounds against 3C-like protease (3CLpro) of SARS-CoV-2 using deep learning and molecular dynamics simulation. *Mol Divers* .2022;26(4) : 10.1007/s11030-021-10330-3 2243-2256 10.1007/s11030-021-10330-3 34637068 PMC8506074

# Response

**Published:** 2024-10-03

**Authors:** Miguel Angel Chavez-Fumagalli

**Affiliations:** Universidad Catolica de Santa Maria, Peru

The research article entitled “Identification of compounds from natural Peruvian sources as potential inhibitors of SARS-CoV-2 Mpro mutations by virtual screening and computational simulations” has found rutin is a potential inhibitor against SARS-CoV-2 Mpro mutations. The research topic is interesting. There are recommendations for the authors to make the manuscript more effective for readers. The manuscript can be considered for indexing on addressing the following major comments.

 The abstract conclusion is not that effective it could be more effective. It should not start with “his” Kindly rephrase it.

**Answer: The abstract was improved**

 The abstract is not very effective kindly re-write it. Kindly refer to these research papers for better understanding Mathpal S, et.al., 2022 (Ref 1) and Joshi T, et.al., 2022 (Ref 2) and cite them accordingly.

**Answer: The abstract was improved**

 The authors have not analyzed the properties of mutations either these are deleterious in nature or neutral mutation. There are many online servers like Meta-SNP. Authors can use these servers.

**Answer: A mutation analysis was done, this was incorporated on Page 4**

 The authors have not analyzed the ADMET and Lipinski properties of compounds. Kindly include these methods.

**Answer: Information was added on Page 5 and Page 6 from the “Virtual Screening analysis” subsection.**

 In the results, the authors have written that mutation is collected from different sources. The short part should be written on the material method and a long can be written on the discussion part.

**Answer: Information was added on page 9. From the “Discussion” section.**

 The results of RMSD are good but for better understanding kindly analyze interaction energy.

**Answer: Information was added on page 9. From the “Discussion” section.**

 The authors have been unable to explain how this study is different from other studies that have also found rutin against Mpro.

**Answer: Information was added on page 9. From the “Discussion” section.**

 There are many loopholes in this study.

**Answer: The manuscript was revised and improved**

 The discussion and conclusion are also not written perfectly. Kindly try to cite recent publications.

**Answer: The manuscript was revised and improved**

 There is a high recommendation for authors to use professional software or language experts to correct the grammar and language of the manuscript. There are many places where sentences look incomplete and create confusion for readers.

**Answer: The manuscript was revised and improved**
